# The German National Cohort (NAKO): Overview and Current State: Karin Halina Greiser

**DOI:** 10.1093/eurpub/ckac129.026

**Published:** 2022-10-25

**Authors:** KH Greiser, B Bohn, L Panreck, E Breunig, W Lieb, T Pischon, T Schikowski, H Völzke

**Affiliations:** 1 Department of Cancer Epidemiology, German Cancer Research Center, Heidelberg, Germany; 2 NAKO e.V., Heidelberg, Germany; 3 NCT Coordination Office, German Cancer Research Center, Heidelberg, Germany; 4 Institute of Epidemiology, Christian-Albrechts-University Kiel, Kiel, Germany; 5 Max Delbrück Center for Molecular Medicine, Berlin, Germany; 6 IUF - Leibniz Institute for Environmental Medicine, Düsseldorf, Germany; 7 Institute for Community Medicine, University Medicine Greifswald, Greifswald, Germany

## Abstract

The presentation aims to give an overview of design, methods and first results of the German National Cohort (NAKO), the largest cohort study in Germany. NAKO is a multidisciplinary, population-based cohort study that provides a central resource for population-based epidemiologic research. NAKO aims to investigate the development and aetiology of diseases, identify risk factors and enhance early detection and prevention of diseases with a focus on diabetes, cancer, cardiovascular, pulmonary, neuropsychiatric and infectious diseases. Between 2014 and 2019, a total of 205,415 persons aged 20 - 74 years were recruited and examined at 18 study centres across Germany. The participants were invited to their local study centre to participate in a face-to-face interview, complete self-administered computer-based questionnaires, undergo a battery of biomedical examinations, and provide various biosamples. In addition, whole-body Magnet Resonance Imaging (MRI) was performed in 30,861 participants on dedicated 3 Tesla MRI scanners at 5 study centres. In 4-5 year intervals, all study participants are re-invited for examinations at the study centres. The programme for the first re-examination (including MRI scanning) was similar to the baseline programme. Thereby, longitudinal information on changes in risk factor profiles and in vascular, cardiac, metabolic, neurocognitive, pulmonary and sensory function is collected. During the COVID-19 pandemic, questions on pandemic-related aspects including the history of infection, severity and long-term health impacts of COVID-19 were added to the examination programme. Since October 2018, 77,896 participants have been re-examined, including 11,382 with additional MRI examination. A supplemental COVID-19 questionnaire was completed by 161,849 participants of NAKO during the first COVID-19-related lockdown in Spring 2020.

